# Does body image dissatisfaction exacerbate smartphone addiction among Chinese college students?

**DOI:** 10.3389/fpsyg.2025.1618979

**Published:** 2025-10-16

**Authors:** Peizhe Xu, Kaichao Shao

**Affiliations:** ^1^Institute of Food Economics, Nanjing University of Finance and Economics, Nanjing, Jiangsu, China; ^2^School of Finance and Accounting, Yellow River Conservancy Technical University, Kaifeng, Henan, China

**Keywords:** smartphone addiction, college students, body image dissatisfaction, APP, gender

## Abstract

**Introduction:**

In the context of the widespread use of mobile internet and smartphones, the issue of excessive smartphone uses and even addiction among college students has become an important social concern. However, the relationship between body image satisfaction, which is a key factor influencing psychological state and behavioral choices, and smartphone addiction has not been fully explored.

**Methods:**

This paper focuses on the impact of college students’ body image satisfaction on smartphone addiction. In the theoretical part, this paper constructs a theoretical framework of smartphone addiction based on objectification theory. Using survey data from 1,958 college students in 7 universities across 3 provinces in China, this study evaluates the levels of smartphone addiction and body image satisfaction among Chinese college students using the Smartphone Addiction Scale-Short Version (SAS-SV) and the Body Image State Scale (BISS). In the empirical part, an OLS model is employed to analyze the impact of college students’ body image satisfaction on smartphone addiction.

**Results:**

The study finds that a 1% decrease in college students’ body image satisfaction leads to a 5.76% increase in smartphone addiction.

**Discussion:**

This conclusion varies by gender and discipline: in terms of gender differences, the impact of body image dissatisfaction on smartphone addiction among female college students is 1% higher than that among male college students; regarding discipline differences, the impact of body image dissatisfaction on smartphone addiction among students in humanities and social sciences is 2% higher than that among students in science and engineering. In the mechanism analysis, it is found that the frequent use of short-video and e-shopping apps among college students further exacerbates the impact of body image dissatisfaction on smartphone addiction.

## 1 Introduction

All individuals possess an appreciation for beauty. Teenagers typically exhibit greater concern for their body image compared to other age demographics (Brasil et al., 2024). This is partly due to the significant alterations in the neuroendocrine system during young adulthood, which propel rapid physical development in teens and heighten their sensitivity to changes in their body image (Blakemore, 2008; Yurgelun-Todd, 2007). On the other hand, as young adults develop psychologically, the imperative to establish a sense of self-identity intensifies. This leads them to focus more on their body image and strive to express their individuality through external representations, thereby attaining identity acknowledgment and self-definition (Branje et al., 2021; Pfeifer and Berkman, 2018). Young adults are increasingly focused on their body image and are endeavoring to cultivate and improve their self-image through numerous methods (Carlson Jones, 2004; Hartman-Munick et al., 2020). Nevertheless, the relentless quest for an ideal body image has exacerbated their discontent with their physical looks (Bassett-Gunter et al., 2017; Grabe et al., 2008). The conflict between the quest for beauty and the apprehension regarding one’s body image has increasingly emerged as a significant issue influencing the mental health of young adults.

Body image dissatisfaction has resulted in various mental health issues among adolescents, including depression (Paxton et al., 2006), body dysmorphia (Pavan et al., 2008), social anxiety (Levinson and Rodebaugh, 2012) and eating disorders (Stice and Shaw, 2002). It has also compelled young adults to adopt diverse strategies to mitigate their worry. The predominant method is objectifying anxiety, namely by seeking psychological solace through dependence on external factors (Slater and Tiggemann, 2010). For instance, engaging in purchasing to mitigate discontent with one’s looks and psychological distress (Tiggemann and Lacey, 2009). Engaging in social interactions to elevate self-esteem through recognition and focus (Cohen and Blaszczynski, 2015).

With the swift advancement of mobile communication technology, the pervasive utilization of smartphones, and the diversification of content, an increasing number of young adults, dissatisfied with their body image, perceive smartphones as a means to alleviate anxiety, thereby intensifying their addiction to these devices (Aljomaa et al., 2016; Roberts et al., 2014). Although the prevalence of smartphone addiction among young adults is widespread, many utilize these devices to escape their body-image dissatisfaction. Empirical analyses examining the impact of body image dissatisfaction on smartphone addiction remain absent. Understanding the scale and mechanisms by which young adults’ body image dissatisfaction influences smartphone addiction is crucial for guiding them in effectively addressing both their body image concerns and addictive behaviors.

Smartphone addiction is characterized as a behavioral addiction marked by persistent cravings, heightened tolerance, and withdrawal symptoms (Billieux et al., 2015; Elhai et al., 2017; Nayak, 2018). Its primary manifestation involves individuals forming an excessive emotional reliance on smartphones and lacking effective self-regulation mechanisms (Li et al., 2020; Xiao et al., 2022). Currently, research on smartphone addiction predominantly concentrates on four key aspects. Firstly, the assessment of smartphone addiction and its underlying causes from a statistical standpoint indicates that loneliness and social anxiety are significant contributors to this phenomenon (Griffiths et al., 2016; Rathakrishnan et al., 2021; Twenge and Campbell, 2018). Secondly, from the standpoint of psychological therapy, it examines the methods for implementing effective interventions and treatments for smartphone addiction. For instance, Cognitive behavioral therapy significantly reduces smartphone addiction (Lian et al., 2016; Yuchang et al., 2017). The third part pertains to social investigations of smartphone addiction. For example, familial context and social support positively influence smartphone addiction (Enez Darcin et al., 2016; Yang et al., 2016). Fourthly, from a technological standpoint, it is essential to examine the impact of elements such as smartphone design and usage behaviors on user addiction, including push notifications and software design (Gómez-Cambronero et al., 2024; Miralles et al., 2020; Ratan et al., 2021).

The current research on smartphone addiction provides a solid foundation for the present study. Nevertheless, three important gaps in the literature merit attention.

1. Empirical research exploring the relationship between body image dissatisfaction and smartphone addiction remains limited. As individuals mature, concerns about body image often become more pronounced. A systematic investigation into the nature of the association between body image dissatisfaction and smartphone addiction is therefore a priority.
2. There is a lack of an integrative theoretical framework for understanding how body image dissatisfaction might relate to smartphone addiction. Existing studies in this area have primarily drawn on compensation theory. In reality, the link between these constructs is likely shaped by a variety of factors. Consequently, reliance solely on the compensation hypothesis to interpret the relationship may oversimplify its complexity.
3. Knowledge of potential heterogeneity and the mechanisms underlying the relationship between body image dissatisfaction and smartphone addiction remains limited. The present study examines how body image dissatisfaction might be associated with smartphone addiction, by considering potential differences across genders and academic disciplines, as well as by exploring possible moderating mechanisms. The findings are expected to offer insights for supporting young adults in managing both body image concerns and smartphone use.

This research administers a questionnaire survey to randomly assess body image satisfaction and smartphone addiction among 1,958 college students from seven colleges in China, considering the previously described difficulties. The OLS model is employed to examine the impact of body image dissatisfaction on smartphone addiction among college students and its underlying mechanism. The innovative characteristics of this work mostly reside in three areas. Firstly, this paper grounded in objectification theory, establishes an analytical framework to examine the correlation between young adults’ dissatisfaction with their body image and smartphone addiction, thereby offering a novel theoretical foundation for comprehending the psychological mechanisms underlying behavioral addiction in young adults and expanding the research scope within this domain. Secondly, this paper employs empirical analysis to comprehensively examine the magnitude and mechanisms by which young adults’ unhappiness with their body image influences smartphone addiction, considering both body image dissatisfaction and body shape dissatisfaction. Thirdly, this research examines the moderating mechanism of mobile app usage in relation to the impact of body image dissatisfaction on smartphone addiction among college students, thus providing a practical basis for formulating effective intervention strategies.

## 2 Theoretical framework and research hypotheses

### 2.1 Boy image dissatisfaction and smartphone addiction

The Objectification Theory asserts that individuals, influenced by their social and cultural milieu, perceive their bodies from an external perspective and consider themselves as objects subject to others’ scrutiny. This results in self-objectification. Individuals experiencing Self-Objectification will progressively cultivate body monitoring. They automatically concentrate on their body image, perpetually concerned about whether their image aligns with societal esthetic standards (Fredrickson and Roberts, 1997). Prolonged exposure to social judgment and comparison can lead individuals to experience shame and dissatisfaction over their body image. Such adverse emotions would exacerbate anxiety in individuals, consequently adversely affecting their mental health (Choma et al., 2009). This psychological imbalance creates conditions conducive to smartphone addiction among young adults (Jones and Griffiths, 2015).

According to the theory of self-objectification, young adults are in a pivotal phase for developing their self-identity. Their self-cognition system is immature, and their self-image formation is significantly reliant on external reinforcement (Adams et al., 2017). In this situation, young adults are inclined to regard others’ assessments of their body image as a significant benchmark, fostering body surveillance. When their look deviates from societal esthetic norms, individuals will encounter body shame and dissatisfaction, therefore eliciting anxiety (Brasil et al., 2024; Saunders et al., 2024). To effectively mitigate anxiety, young adults frequently engage in self-regulation methods that are seamlessly incorporated into their daily routines and easily managed autonomously, such as virtual social interactions and online gaming (Rotondi et al., 2017). Smartphones, characterized by portability, immediate feedback, abundant information, and varied interaction, effectively meet the psychological adjustment requirements of young adults and have emerged as their favored option for mitigating anxiety (Aljomaa et al., 2016; Fardouly et al., 2015; Roberts et al., 2014). The primary reasons young adult’s self-objectification fosters smartphone addiction are threefold:

A primary reason for young adults’ addiction to smartphones is the rapid reward system involving dopamine in the brain. Social media services, like post likes and brief video clips, stimulate the brain’s reward system through constant engagement, offering young adults rapid gratification and momentarily mitigating their discontent with their body image (O’Day and Heimberg, 2021).

A second is the augmenting impact of “virtual self” identification. Young adults utilize smartphones to create “digital identities” as a means of evading discontent with their body image, thus establishing a “circular process of objectification of the virtual self” (Suh, 2013). As the scrutiny of their physical bodies increases, anxiety correspondingly escalates.

A third is the impact of escapism. Young adults replace genuine interpersonal engagement with virtual social interactions to evade potential negative assessments related to their body image in real life. They transform their smartphones into a sanctuary to evade the pressures of their body image in reality (Hu et al., 2022).

Following the preceding study, this paper proposes Hypothesis 1:

*H1*: A negative self-image among young adults increases their susceptibility to smartphone addiction.

### 2.2 Gender and discipline-wise variations in smartphone addiction

Smartphone addiction is characterized by psychological addiction, defined as a condition in which an individual exhibits a profound dependence and lacks control over specific behaviors or substances (Wise and Koob, 2014). Initial studies on addiction mostly concentrated on substance dependence, including drug and alcohol addiction (Wagner and Anthony, 2002). As non-material addictive behaviors have proliferated, researchers have started to focus on behavioral addictions, including gambling and gaming (Wanigaratne, 2006). Addiction psychology posits that addictive behaviors are linked not only to physiological mechanisms but also to an individual’s psychological condition and social context (Nutt et al., 2015). The pervasive use of the Internet and smartphones has led to smartphone addiction becoming a prevalent issue among today’s young adults (De-Sola Gutiérrez et al., 2016; Marengo et al., 2022). “Smartphone addiction” denotes a psychological illness characterized by excessive reliance on smartphones, leading to substantial effects on everyday activities and professional responsibilities (Salicetia, 2015; Sapacz et al., 2016). The study found that smartphone addiction not only differs by gender but also varies with an individual’s level of education (Alhassan et al., 2018; Chóliz, 2010).

From the standpoint of gender disparities, females are more prone to “self-objectification,” which subsequently results in body monitoring. Females who are unsatisfied with their body image tend to increase their smartphone usage for body-image management and grooming, striving to conform to societal esthetic norms (Fardouly and Vartanian, 2016). Moreover, when females are significantly distressed by their body image, they are more likely to engage with social media to obtain social validation and emotional support, thus mitigating their anxiety (Vandenbosch et al., 2022). This form of social compensatory behavior not only extends smartphone usage but may also increase the risk of smartphone addiction by strengthening psychological dependence on virtual interactions. In comparison to the female cohort, males typically exhibit diminished care for esthetics. Males often resort to exercise as a coping mechanism when dissatisfied with their body image (Burnette et al., 2017). Consequently, in terms of discontent with body image, the prevalence of smartphone usage and the risk of addiction among male students are significantly lower than those among female students.

From the standpoint of discipline-wise variation, college students specializing in humanities and social sciences exhibit a significant reliance on digital resources throughout their academic pursuits and daily lives. During the learning process, individuals must engage with literature, compose papers, and participate in online debates and exchanges (Kubey et al., 2001; Stellner and Vokoun, 2014). The intrinsic digital requirements of this field have resulted in an increased propensity among students majoring in humanities and social sciences to utilize smartphones. In contrast to students pursuing humanities and social sciences (HSS), those studying science and engineering (SE) depend more on experiments and practical applications for learning and communication, exhibiting reduced reliance on the Internet (Hargis, 2001; Lukychova et al., 2022). Moreover, the study schedules of scientific and engineering students are quite constrained, and the fragmented internet time they can allocate is similarly restricted. This effectively diminishes the likelihood of excessive smartphone usage (Mella-Norambuena et al., 2021). Considering the above-described distinctions, this study proposes the following hypotheses:

*H2a*: The impact of body image dissatisfaction on smartphone addiction in female students exceeds that in male students.

*H2b*: The impact of dissatisfaction with body image on smartphone addiction is more pronounced among students of humanities and social sciences than among students of science and engineering.

### 2.3 The moderating effect of APP

Based on the functional properties of mobile applications (APPs), APPs significantly moderate the association between body image dissatisfaction and smartphone addiction among college students. The varied functions of applications, including social interaction, entertainment, purchasing, information retrieval, and education, offer digital platforms for college students to articulate their interests and fulfill their unique requirements. Nonetheless, these apps heighten the danger of smartphone addiction due to amplified behaviors (Bopp et al., 2016; Lin et al., 2015). The frequent utilization of apps and the proliferation of their content will exacerbate the addictive effect, particularly when college students are dissatisfied with their body image and are in pursuit of external validation or psychological compensation (Pryde and Prichard, 2022). Consequently, comprehending the app’s moderating effect is essential for exploring the impact of body image dissatisfaction on smartphone usage behavior (Embacher Martin et al., 2018).

From a social interaction standpoint, young adults with elevated social engagement typically utilize social applications more often, and their online social conduct is marked by frequent interactions and intentional body image curation. The digital portrayal of body image is more likely to incite “self-objectification” in adolescents, exacerbating anxiety related to body image dissatisfaction and heightening the likelihood of smartphone addiction (Oliveira et al., 2021; Yang et al., 2022).

Considering the attributes of short-video applications, young adults are more inclined to be captivated by fragmented and intensely engaging films that have numerous transitions. This pattern of information consumption may reinforce the addiction process in young adults (Qin et al., 2022). When individuals perceive a dissonance between their “body” and “society,” they tend to increase their smartphone usage to evade “body surveillance,” thus exacerbating their behavioral reliance on smartphone addiction (Liu et al., 2020).

Additionally, certain applications are engineered to augment users’ physical capabilities. Nevertheless, if they are excessively relied upon or misapplied, they may exacerbate individuals’ discontent with their physical flaws, consequently heightening their worry over body image and fostering an obsessive quest for an ideal physique (Robinson et al., 2017).

In contrast to visually-oriented applications, non-visual apps like music, reading, and e-shopping exhibit diminished engagement with body image representation, resulting in a reduced capacity to elicit body shame. Their advocacy for smartphone addiction is likewise somewhat restricted. It is noteworthy that, although these applications are not directly associated with bodily cognition, they may alleviate real-life stress through immersive experiences. Excessive use may still result in behavioral dependence (Linnemann et al., 2016). Various sorts of applications exert distinct influences on the relationship between body image dissatisfaction and smartphone addiction. This study posits Hypothesis 3 based on the preceding analysis.

*H3*: Significant variances exist in the Moderating Effect of various types of apps. The moderating effect of visual applications is substantially greater than that of non-visual applications.

## 3 Research design

### 3.1 Background and data

With the advancement of mobile information technology, smartphones have been intricately woven into daily life, serving as the primary conduit for users to engage with the external world. The deep integration of smartphones into daily life is particularly pronounced in China. International Data Corporation (IDC) reports that from 2014 to 2023, mobile internet users in China increased from 464 million to 1.079 billion, reflecting an average annual compound growth rate of 9.3%. Including over 80% of the population. The smartphone penetration rate among college students, as a digital native demographic, surpasses 98%. According to data from the China Internet Network Information Center (CNNIC), more than 70% of college students utilize their smartphones for over 4 h each day, with 29.7% exceeding 6 h, and 53.2% of participants self-reporting varied levels of smartphone addiction. The statistics suggest that the overuse of smartphones has emerged as a significant societal issue among college students.

This study aims to explore the relationship between appearance satisfaction and smartphone addiction tendencies among Chinese college students. The study subjects are students currently enrolled in Chinese universities, with samples drawn from seven universities in three cities (Zhengzhou, Kaifeng, and Nanjing) with high population densities in Henan and Jiangsu provinces, covering both provincial capitals and cities with local characteristics. After obtaining informed consent from the participating schools and students themselves, the survey was administered in questionnaire form. The research instruments included: the validated body image Satisfaction Scale (Body Image State Scale, BISS) developed by Cash et al. (2002) to assess body image satisfaction; and the Smartphone Addiction Scale–Short Version (SAS-SV) developed by Kwon et al. (2013) to assess smartphone addiction tendencies. Additionally, a variable questionnaire containing information on gender, age, grade level, and other demographics was used. All participants in this study received a detailed explanation on the first page of the questionnaire regarding the purpose of the study, the use of data, the voluntary nature of participation, and measures for anonymity and confidentiality. They began answering the questions only after confirming their consent. All data were collected and stored anonymously, to be used solely for this study and for no other purposes. The specific operations are as follows:

1. Sampling stratification. Taking the grade attribute of the research subjects as the criterion, it covers undergraduate (freshman to senior) and postgraduate (master’s and doctoral) stages, with a total of 6 independent strata. Stratification can avoid excessive concentration of samples from a single academic stage.
2. Randomization implementation. Conducted in three steps on the premise of students’ voluntary participation: Step 1, Informed consent and registration. After obtaining the informed consent of the participating schools and students, the voluntary registration information of students in each grade is collected, and a unique ID is assigned to each registrant; Step 2, Random sampling. A unique random number between 0 and 1 is generated for all voluntary participants, and its uniform distribution characteristic is used to ensure that each student has an equal probability of being selected; Step 3, Stratified sampling and sample integration. Participants are ranked according to the random numbers, and the number of samples to be selected from each stratum is determined based on the total sample size and the proportion of each grade. Finally, the samples are merged to form 1,998 initial samples. After screening for questionnaire completeness, 1,958 valid samples are obtained.

### 3.2 Definition of variables

#### 3.2.1 Smartphone addiction

This study employed the Smartphone Addiction Scale–Short Version (SAS-SV) to assess the degree of smartphone addiction among adolescents. Developed by Kwon et al. (2013) based on the original 33-item Smartphone Addiction Scale (SAS), the SAS-SV was streamlined to retain 10 core items, making it suitable for measuring smartphone addiction levels in adolescents (See Table 1).

**Table 1:** The SAS-SV includes content.

Code	Definitions	Scoring
1	Missing planned work due to smartphone use	1–6
2	Having a hard time concentrating in class, while doing assignments, or while working due to smartphone use	1–6
3	Feeling pain in the wrists or at the back of the neck while using a smartphone	1–6
4	Won’t be able to stand not having a smartphone	1–6
5	Feeling impatient and fretful when I am not holding my smartphone	1–6
6	Having my smartphone in my mind even when I am not using it	1–6
7	I will never give up using my smartphone even when my daily life is already greatly affected by it.	1–6
8	Constantly checking my smartphone so as not to miss conversations between other people on Twitter or Facebook	1–6
9	Using my smartphone longer than I had intended	1–6
10	The people around me tell me that I use my smartphone too much.	1–6

The SAS-SV is constructed around the core characteristics of smartphone addiction, covering six dimensions: (1) interference with daily life, (2) positive anticipation, (3) withdrawal symptoms, (4) cyberspace-oriented relationships, (5) overuse and tolerance, and (6) emotional relief. The scale uses a 5-point Likert scoring method, with respondents rating each item from “1 = strongly disagree” to “5 = strongly agree” based on their own experiences. The total score is obtained by summing the scores of the 10 items, with higher total scores indicating a more pronounced tendency toward smartphone addiction.

To verify the applicability of the scale in this study’s sample, reliability and validity tests were conducted. The results showed that the internal consistency coefficient Cronbach’s *α* was 0.844, which is well above the acceptable standard of 0.7, indicating good homogeneity among the scale items and excellent reliability. The KMO measure of sampling adequacy was 0.738, exceeding the critical value of 0.7, and the Bartlett’s test of sphericity was significant, indicating that the data were suitable for exploratory factor analysis. The scale’s structural validity met the research requirements and can be used to measure the smartphone addiction variable in this study.

#### 3.2.2 Body image satisfaction

This study employed the BISS for measurement. The Body Image States Scale (BISS) is a state-specific body image scale developed by Cash et al. (2002), used to rapidly assess an individual’s immediate feelings and satisfaction with their physical body image at a specific point in time. The BISS is a self-report scale consisting of six items, scored on a 9-point Likert scale, with higher scores indicating higher body image satisfaction (See Table 2). The specific items include: “At this moment, I am satisfied with my physical appearance”; “Right now, I find my body attractive”; “Currently, I feel confident about my body”; “At present, I feel good about my physical appearance”; “At this time, I have a positive body image”; “Right now, I am proud of my body.” All items are scored on a 9-point Likert scale (1 = strongly disagree, 9 = strongly agree), with a total score ranging from 6 to 54. Higher scores indicate higher appearance satisfaction at the time of measurement.

**Table 2:** The BISS includes content.

Code	Definitions	Scoring
1	Right now, I feel (Extremely dissatisfied to Extremely satisfied) with my physical appearance.	1–9
2	Right now, I feel (Extremely dissatisfied to Extremely satisfied) with my body size and shape.	1–9
3	Right now, I feel (Extremely dissatisfied to Extremely satisfied) with my weight.	1–9
4	Right now, I feel (Extremely physically unattractive to Extremely physically attractive).	1–9
5	Right now, I feel (Much worse to Much better) than I usually feel about my appearance.	1–9
6	Right now, I feel (Much worse to Much better) than the average person looks.	1–9

To verify the applicability of the scale in this study’s sample, reliability and validity tests were conducted. The results showed that the internal consistency coefficient Cronbach’s α was 0.826, which is well above the acceptable standard of 0.7, indicating good homogeneity among the scale items and excellent reliability. The KMO measure of sampling adequacy was 0.776, exceeding the critical value of 0.7, suggesting that the data is suitable for factor analysis. The Bartlett’s test of sphericity was significant, indicating significant correlations among the items. Overall, the BISS scale is suitable for measuring appearance satisfaction among adolescents in Chinese universities in this study.

According to the aforementioned research findings, dissatisfaction with personal body image may intensify college students’ dependence on smartphones. Pertinent studies further demonstrate that such dissatisfaction significantly influences their smartphone usage behavior. For example, Holland and Tiggemann (2016) conducted a study revealing that anxiety regarding body image prompts individuals to engage more frequently with social media in pursuit of recognition and validation from others. This behavioral pattern exacerbates internet addiction.

#### 3.2.3 Moderating variables

We consider the sorts of smartphone applications often utilized by college students as the moderating variable. They are categorized into eight classifications based on their functional attributes: social interaction, short video, shopping, life services, mobile games, news and information, e-reading, and mobile video. The study statistically measures the frequency and duration of application usage across two dimensions via structured questionnaires, employing binary variable assignment (0 = infrequently used, 1 = frequently used) to ascertain the usage status of the programs (Table 3).

**Table 3:** Definition and assignment of variables.

Variables	Definition and assignment
Smartphone addiction	SAS-SA
Smartphone usage time	The daily duration of smartphone usage (in hours).
Body image satisfaction	BISS
Gender	Male or female: Female = 0; Male = 1.
Age	Age range: 18–26 years old.
Height	What’s your height (cm)?
Weight	What’s your weight (kg)?
Discipline	The discipline of your major: HSS = 0; SE = 1.
Grade	Your academic level: From freshman year to doctoral candidate.
Grades	Ranging from poor to excellent, are assigned values from 1 to 5.
Social media APP	Whether you use smartphones for social communication for a long time every day: No = 0; Yes = 1.
Short videos APP	Whether you use smartphone short-video apps for a long time every day: No = 0; Yes = 1.
E-shopping APP	Whether you use smartphone for shopping apps for a long time every day: No = 0; Yes = 1.
Life APP	Whether you use smartphone life apps for a long time every day: No = 0; Yes = 1.
Games APP	Whether you use smartphone games for a long time every day: No = 0; Yes = 1.
News App	Whether you use smartphone news apps for a long time every day: No = 0; Yes = 1.
E-book APP	Whether you use smartphones or e-book apps for a long time every day: No = 0; Yes = 1.
Video APP	Whether you use smartphone video apps for a long time every day: No = 0; Yes = 1.

#### 3.2.4 Control variables

To guarantee the precision and dependability of the research findings, the following control variables were incorporated in this study: gender (male or female), age (determined by particular ages), height (measured in meters), and weight (measured in kilograms). Furthermore, we considered the potential effects of subject type and grade on the research.

### 3.3 Statistical analysis of data

To clarify the basic distribution characteristics of the data, this study conducted descriptive statistical analysis on each core variable, with the results shown in Table 4. In terms of smartphone addiction levels, the average score for college students’ smartphone addiction, measured by the SAS-SV scale (with a total score range of 10–60 points), was 30.71, with a standard deviation of 7.82. The minimum value was 12 and the maximum value was 54, indicating significant individual differences in smartphone addiction levels among the college students in the sample, with an overall moderate-to-high level. Regarding smartphone usage time, the average daily smartphone usage time for college students was 5.62 h, with a standard deviation of 1.88, reflecting a relatively high intensity of smartphone use among current college students. In the dimension of appearance satisfaction, the average score for college students’ appearance satisfaction, measured by the BISS scale (with a total score range of 6–54 points), was 27.94 with a standard deviation of 10.45. The minimum value was 6 and the maximum value was 52, indicating that the overall appearance satisfaction of the sample was at a moderate-to-low level, with significant group heterogeneity.

**Table 4:** Statistical analysis.

Variables	Obs.	Mean	Std. Dev.	Min	Max
SAS-SV	1,958	30.71	7.82	12	54
Smartphone usage time	1,958	5.62	1.88	1	8
BISS	1,958	27.94	10.45	6	52
Grades	1,958	3.47	3.15	1	5
Gender	1,958	0.61	0.48	0	1
Age	1,958	19.14	1.27	18	26
Height	1,958	168.89	8.84	148	200
Weight	1,958	62.52	14.01	38	130
Discipline	1,958	1.37	0.48	1	2
Grade	1,958	1.41	0.87	1	6
Social media APP	1,958	0.92	0.26	0	1
Short-videos APP	1,958	0.77	0.42	0	1
E-shopping APP	1,958	0.54	0.49	0	1
Life APP	1,958	0.38	0.48	0	1
Games APP	1,958	0.36	0.48	0	1
News App	1,958	0.16	0.36	0	1
E-book APP	1,958	0.28	0.45	0	1
Video APP	1,958	0.36	0.48	0	1

Variables including Social Media APP, Short-video APP, E-shopping APP, Life APP, Games APP, News APP, E-book APP, and Video APP are binary variables. The assignment rule is: 1 = “use the APP for a long time every day” (defined as daily usage duration≥3 h, consistent with the operational definition in the questionnaire), 0 = “do not use the APP for a long time every day”.

### 3.4 Correlation analysis of data

This study utilized Pearson correlation analysis to ass ess multicollinearity. The findings indicated (see Table 5) that the highest correlation coefficient among the variables was 0.6482, which was markedly below the essential threshold of 0.8. This signifies the absence of substantial multicollinearity issues in the data, establishing a dependable basis for the next study.

**Table 5:** Correlation analysis.

Variables	BISS	Gender	Age	Height	Weight	Grades	Discipline	Grade
BISS	1							
Gender	−0.0153	1						
Age	0.0429	0.0145	1					
Height	0.1006	−0.6482	−0.0079	1				
Weight	−0.0297	−0.5278	0.031	0.5674	1			
Grades	−0.0163	−0.0046	−0.016	−0.331	0.07	1		
Discipline	0.0141	−0.2462	−0.0571	0.1851	0.1529	0.0355	1	
Grade	0.0498	0.1218	0.7862	−0.0637	−0.0292	−0.0102	−0.1237	1

This table presents the results of correlation analysis using Pearson correlation coefficients. The maximum correlation coefficient between variables is 0.6482, which is below the commonly accepted threshold of 0.8, indicating no significant multicollinearity. Additionally, the variance inflation factor (VIF) for all variables is less than 10, further confirming that the data meets the assumption of no multicollinearity required for OLS regression.

### 3.5 Model

To study the influence of dissatisfaction with one’s body image on smartphone addiction among college students, this paper designs an OLS econometric model for empirical analysis. The specific model is as follows Equation (1):


(1) $${\mathrm{SAS}}_{i}=\alpha_{0}+\alpha_{1}{\text{BISS}}_{i}+\alpha_{2}\sum {\text{Control}}_{i}+\lambda_{j}+\varepsilon_{i}$$

Herein, SAS symbolizes the degree of college students’ smartphone addiction. The sign “*i”* indicates an individual. BISS implies Body Image satisfaction. C*ontrol* represents the control variables for individuals, which specifically include the individual’s gender, age, height, weight, grade, Discipline, etc. α represents the parameter to be estimated. $\lambda_{j}$ denotes a provincial fixed impact. ε denotes the stochastic error component.

To further examine the moderating influence of mobile applications on the correlation between body image satisfaction and smartphone addiction, we established the subsequent model based on Equation (2):


(2) $$\begin{array}{l} {\mathrm{SAS}}_{i}=\alpha_{0}+\alpha_{1}{\text{BISS}}_{i}+\alpha_{2}{\mathrm{APP}}_{i}+\alpha_{3}{\text{BISS}}_{i}\cdot {\mathrm{App}}_{i}+\\ \alpha_{4}\sum {\text{Control}}_{i}+\lambda_{j}+\varepsilon_{i} \end{array}$$

## 4 Empirical analysis

### 4.1 Baseline regression

Table 6 presents the benchmark regression analysis results on the impact of body image satisfaction on smartphone addiction among college students. Models (1) to (3) progressively incorporate control variables to test the robustness of the results. Model (1), which does not include any control variables and does not control for regional fixed effects, shows a regression coefficient of −0.0611 for body image satisfaction, significant at the 1% statistical level. This indicates that, without considering other confounding factors, a 1% decrease in body image satisfaction is associated with a 6.11% increase in smartphone addiction among college students. In Model (2), after incorporating control variables such as individual characteristics, the regression coefficient for body image satisfaction becomes −0.0577, still significant at the 1% level, with a slight decrease in the absolute value of the coefficient. This suggests that, after controlling for individual-level differences, a 1% decrease in body image satisfaction is associated with a 5.77% increase in smartphone addiction. Model (3), which includes control variables and controls for regional effects, shows a regression coefficient of −0.0576 for body image satisfaction, significant at the 5% level. This indicates that a 1% decrease in appearance satisfaction is associated with a 5.76% increase in smartphone addiction. The study results show a significant negative correlation between body image satisfaction and smartphone addiction among college students, meaning that lower body image satisfaction is associated with higher smartphone addiction, thus validating Hypothesis 1.

**Table 6:** Baseline regression results.

Variables	(1)	(2)	(3)
BISS	−0.0611^***^ (0.0168)	−0.0577^***^ (0.0173)	−0.0576^***^ (0.0178)
Control variables	No	Yes	Yes
Region	No	No	Yes
Individual	No	No	Yes
*N*	1,958	1,958	1,958
*R* ^2^	0.007	0.021	0.049

(1) The estimated results in the table are obtained through OLS model regression. (2) Model (1) presents the regression results without including control variables, regional effects, and individual effects. Model (2) shows the regression results with control variables included but without controlling for regional and individual effects. Model (3) displays the regression results with control variables, regional effects, and individual effects all included. (3) From the results, the regression results of Models (1) to (3) are all significant. As the model controls become more stringent, the absolute values of the regression coefficients decrease. (4) According to the results of Model (3), for every 1% decrease in BISS, smartphone addiction increases by 5.76%. (5) ***, **, and * signify that the estimation findings are statistically significant at the 1, 5, and 10% levels, respectively. (6) The robust standard errors are enclosed in parentheses.

### 4.2 Robustness test

To test the robustness of the OLS regression results, this study conducted two independent tests: first, replacing the OLS regression with the Generalized Least Squares (GLS) regression; second, using smartphone usage time as the core variable to replace the Body Image State Scale (BISS). Through the above tests, the robustness of the baseline regression results was analyzed.

#### 4.2.1 Robustness test: alternative estimation based on generalized least squares (GLS) regression

To mitigate the potential impact of heteroscedasticity in the data on the robustness of the results, this study employed the generalized least squares (GLS) regression model for alternative estimation, with the results shown in Table 7. Model (1) presents the regression results without including control variables or regional fixed effects, showing a BISS regression coefficient of −0.0998, significant at the 1% significance level. Model (2) includes control variables, with the BISS regression coefficient remaining −0.0998 and significant at the 1% level. Model (3) incorporates both control variables and regional fixed effects, yielding a BISS regression coefficient of −0.0981, also significant at the 1% level. This indicates a significant negative correlation between college students’ body image satisfaction and smartphone addiction, meaning that lower body image satisfaction is associated with a higher propensity for smartphone addiction among college students. The above results demonstrate that after correcting for the potential impact of heteroscedasticity using the GLS model, the negative association between body image satisfaction and smartphone addiction among college students remains stable and significant. A 1% decrease in body image satisfaction is associated with a 9.8% increase in smartphone addiction levels, suggesting that college students with lower body image satisfaction have a higher tendency toward smartphone addiction. This conclusion is highly consistent with the benchmark regression results, further validating the robustness of the negative correlation between the two.

**Table 7:** Alternative estimation based on GLS regression.

Variables	(1)	(2)	(3)
BISS	−0.0998*** (0.0005)	−0.0998*** (0.0006)	−0.0981*** (0.0006)
Control variables	No	Yes	Yes
Region	No	No	Yes
Individual	No	No	Yes
*N*	1,958	1,958	1,958

(1) The estimated results in the table are derived from regression analysis using the Generalized Least Squares (GLS) model. (2) Model (1) presents the regression results without incorporating control variables, regional effects, or individual effects. Model (2) shows the regression results with control variables included but without controlling for regional and individual effects. Model (3) displays the regression results with control variables, regional effects, and individual effects all incorporated. (3) From the results, the regression results of Models (1) to (3) are all significant, which indicates that for every 1% decrease in body satisfaction, smartphone addiction increases by 9.8%. ***, **, and * signify that the estimation findings are statistically significant at the 1%, 5%, and 10% levels, respectively.

#### 4.2.2 Robustness test: substituting the dependent variable (with daily smartphone usage duration)

To further verify the robustness of the impact of body image satisfaction on smartphone usage behavior among college students, this study replaced the dependent variable with “smartphone usage time” and conducted the regression analysis again, with the results shown in Table 8. Model (1) presents the regression results without including control variables or regional fixed effects, showing a BISS regression coefficient of −0.0441, significant at the 1% significance level. Model (2) includes control variables, with the BISS regression coefficient being −0.0577 and significant at the 1% level. Model (3) incorporates both control variables and regional fixed effects, yielding a BISS regression coefficient of −0.0576, also significant at the 1% level. This indicates a significant negative correlation between college students’ BISS and smartphone addiction, meaning that lower body image satisfaction is associated with a higher propensity for smartphone addiction among college students. The above results show that regardless of whether individual characteristics and regional variables are controlled, body image satisfaction among college students has a stable and significant negative impact on daily smartphone usage duration, that is, the lower the body image satisfaction, the longer the daily smartphone usage duration. This is consistent with the benchmark regression results.

**Table 8:** Substituting the dependent variable with smartphone usage time.

Variables	(1)	(2)	(3)
	Smartphone usage time	Smartphone usage time	Smartphone usage time
BISS	−0.0441*** (0.0025)	−0.0577*** (0.0173)	−0.0576*** (0.0178)
Control variables	No	Yes	Yes
Region	No	No	Yes
Individual	No	No	Yes
*N*	1,958	1,958	1,958
*R* ^2^	0.002	0.021	0.049

(1) The estimated results in the table are obtained through regression analysis using the OLS model. (2) Model (1) presents the regression results without including control variables, regional effects, and individual effects. Model (2) shows the regression results with control variables included but without controlling for regional and individual effects. Model (3) displays the regression results with control variables, regional effects, and individual effects all incorporated. (3) From the results, the regression results of Models (1) to (3) are all significant, which also indicates that the lower the body satisfaction, the higher the level of smartphone addiction. ***, **, and * signify that the estimation findings are statistically significant at the 1%, 5%, and 10% levels, respectively.

### 4.3 Classification

#### 4.3.1 Gender disparities

To test Hypothesis 2a, this study conducted regression analyses based on gender groups to explore the gender heterogeneity in the relationship between body image satisfaction and smartphone addiction, with the results shown in Table 9.

**Table 9:** Gender disparities in the impact of college students’ body image satisfaction perception on smartphone addiction.

Variables	(1)	(2)	(3)	(4)
	Female	Male	Female	Male
BISS	−0.0641*** (0.0248)	−0.0564** (0.0227)	−0.0653** (0.0269)	−0.0582** (0.0241)
Control variables	No	No	Yes	Yes
Region	No	No	Yes	Yes
Individual	No	No	Yes	Yes
*N*	769	1,179	769	1,179
*R* ^2^	0.008	0.005	0.064	0.050

(1) The estimated results in the table are obtained through regression analysis using the OLS model. (2) Models (1) and (2) present the regression results without including control variables and without controlling for regional effects and individual effects. Models (3) and (4) show the regression results with control variables included and with regional effects and individual effects controlled for. (3) Specifically, Models (1) and (3) display the regression results for female college students, while Models (2) and (4) present those for male college students. (4) From the estimated results, the absolute value of the BISS coefficient for female college students is higher than that for male college students. This indicates that the degree of smartphone addiction caused by body image dissatisfaction among female college students is approximately 1% higher than that among male college students. ***, **, and * signify that the estimation findings are statistically significant at the 1%, 5%, and 10% levels, respectively.

Models (1) and (2) present the gender-specific regression results without including control variables or controlling for regional fixed effects. The results show that the BISS regression coefficients for male and female college students are 0.0564 and 0.0641, respectively, both significant at the 1% significance level. By comparing the absolute values of the coefficients, it can be seen that the absolute value of the coefficient for the female group is 0.0077 higher than that for the male group. This means that for every 1% decrease in body image satisfaction, the increase in smartphone addiction level is 0.77% higher for female students than for male students, equivalent to the negative marginal effect of body image satisfaction on smartphone addiction being approximately 13.65% higher for females than for males.

Models (3) and (4) present the gender-specific regression results after incorporating control variables for individual characteristics and controlling for regional fixed effects. The results show that the regression coefficients for body image satisfaction are −0.0582 for the male group and −0.0653 for the female group, both significant at the 1% statistical level. At this point, the absolute value of the coefficient for the female group is 0.0071 higher than that for the male group. This indicates that after controlling for confounding factors, the negative marginal effect of body image satisfaction on smartphone addiction is still about 12.20% higher for female students than for male students. In other words, when body image satisfaction decreases, the increase in smartphone addiction level is significantly greater for female college students than for male college students.

In summary, whether or not control variables and regional effects are controlled, the negative impact of body image satisfaction on smartphone addiction is stronger for female college students than for male college students. That is, females’ concerns about their body image are more likely to translate into smartphone addictive behavior, which is completely consistent with the expectation of Hypothesis 2a.

#### 4.3.2 Discipline-wise variations

To test Hypothesis 2b, this study conducted group regression analyses based on academic disciplines (Humanities and Social Sciences HSS/Science and Engineering SE) to explore the differences in the relationship between body image satisfaction and smartphone addiction across disciplines, with the results shown in Table 10.

**Table 10:** Discipline-wise variations in the impact of college students’ body image satisfaction perception on smartphone addiction.

Variables	(1)	(2)	(3)	(4)
	HSS	SE	HSS	SE
BISS	−0.0644*** (0.0214)	−0.0542** (0.0272)	−0.0664*** (0.0227)	−0.0445** (0.0187)
Control variables	No	No	Yes	Yes
Region	No	No	Yes	Yes
Individual	No	No	Yes	Yes
*N*	717	1,241	717	1,241
*R* ^2^	0.007	0.005	0.053	0.077

(1) The estimated results in the table are derived from regression analysis using the OLS model. (2) Models (1) and (2) present the regression results without including control variables or controlling for regional and individual effects. Models (3) and (4) show the regression results with control variables included and regional and individual effects controlled for. (3) Among them, Models (1) and (3) display the regression results for students in humanities and social sciences, while Models (2) and (4) present those for students in science and engineering. (4) According to the estimated results, the absolute values of the coefficients for humanities and social sciences students are higher than those for science and engineering students. This indicates that the degree of smartphone addiction caused by body image dissatisfaction among humanities and social sciences college students is approximately 1% higher than that among science and engineering college students. ***, **, and * signify that the estimation findings are statistically significant at the 1%, 5%, and 10% levels, respectively.

Models (1) and (2) present the discipline-specific regression results without including control variables or controlling for regional fixed effects. The results show that in the HSS group, the regression coefficient for BISS on smartphone addiction is −0.0644, significant at the 1% statistical level; in the SE group, the BISS regression coefficient is −0.0542, significant at the 5% statistical level. By comparing the absolute values of the coefficients, it can be seen that the absolute value of the coefficient for the HSS group (0.0644) is 0.0102 higher than that for the SE group (0.0542). This means that for every 1% increase in body image satisfaction, the decrease in smartphone addiction level is 1.02% higher for HSS students than for SE students. Based on the coefficient of the SE group, the negative marginal effect of body image satisfaction on smartphone addiction is 18.82% higher for HSS students than for SE students.

Models (3) and (4) present the discipline-specific regression results after incorporating control variables for individual characteristics and controlling for regional fixed effects. The results show that the regression coefficient for body image satisfaction in the HSS group is −0.0664, significant at the 1% statistical level; in the SE group, the regression coefficient is −0.0445, significant at the 5% statistical level. At this point, the absolute value of the coefficient for the HSS group is 0.0219 higher than that for the SE group. Based on the coefficient of the SE group, the negative marginal effect of body image satisfaction on smartphone addiction is approximately 49.21% greater for HSS students than for SE students. This indicates that after controlling for confounding factors, when body image satisfaction decreases, the increase in smartphone addiction level is significantly greater for HSS students than for SE students, which is completely consistent with the expectation of Hypothesis 2b.

## 5 Mechanism analysis

To test Hypothesis 3, this study categorized the frequently used smartphone apps by college students into eight types, including social media, short video, e-shopping, etc. By constructing interaction terms of “BISS*APP” in the multiple regression models, the moderating effects of different app types were explored, with the results shown in Table 11. Models (1) to (8) correspond to the moderating effect tests of the eight app, respectively, and the specific analyses are as follows:

**Table 11:** Mechanism of APP.

Variables	(1)	(2)	(3)	(4)	(5)	(6)	(7)	(8)
BISS	−0.0595^***^ (0.0171)	−0.0534^***^ (0.0168)	−0.0774^***^ (0.0238)	−0.0776^***^ (0.0213)	−0.0527^**^ (0.0207)	−0.0681^***^ (0.0186)	−0.0613^***^ (0.0198)	−0.0716^***^ (0.0207)
Social media APP	0.2008^***^ (0.0065)							
BISS*social media	−0.0341 (0.0543)							
Short-video APP		0.0971^***^ (0.0133)						
BISS*Short-video		0.3102^***^ (0.0396)						
E-shopping APP			0.2194^***^ (0.0359)					
BISS*e-shopping			0.0725^***^ (0.0119)					
Life APP				0.5758 (0.5988)				
BISS*life				0.0481 (0.0342)				
Gaming APP					0.3051^***^ (0.1007)			
BISS*gaming					0.0051 (0.0338)			
News APP						−0.2421^*^ (0.1367)		
BISS*news						0.0664 (0.0428)		
E-book APP							0.8115 (1.101)	
BISS*e-book							0.0094 (0.0365)	
Video APP								0.9522 (1.0325)
BISS*video								0.0328 (0.0344)
Control variables	Yes	Yes	Yes	Yes	Yes	Yes	Yes	Yes
Region	Yes	Yes	Yes	Yes	Yes	Yes	Yes	Yes
Individual	Yes	Yes	Yes	Yes	Yes	Yes	Yes	Yes
*N*	1,958	1,958	1,958	1,958	1,958	1,958	1,958	1,958

(1) The estimated results in the table are obtained from regression analysis using the Generalized Least Squares (GLS) model. (2) Models (1) to (8) correspond to the regression results of the moderating effects of Social Media APP, Short-video APP, Life APP, Gaming APP, News APP, E-book APP, and Video APP, respectively. (3) Observing the interaction terms, only the moderating effects of Short-video APP and E-shopping APP are relatively significant. ***, **, and * signify that the estimation findings are statistically significant at the 1%, 5%, and 10% levels, respectively.

Social media apps (Model 1): The main effect coefficient for social media app usage is 0.2008, which passes the 1% significance test; however, the interaction term coefficient for “BISS*social media apps” is −0.0341 and does not pass the significance test, indicating that the use of social media apps does not significantly moderate the relationship between body image satisfaction and smartphone addiction.

Short-video apps (Model 2): The main effect coefficient for short video app usage is 0.0971, which passes the 1% significance test; the interaction term coefficient for “BISS*short-video apps” is 0.3102 and also passes the 1% significance test. The positive interaction term coefficient indicates that the use of short video apps plays a positive moderating role.

E-shopping apps (Model 3): The main effect coefficient for e-shopping app usage is 0.2194, which passes the 1% significance test; the interaction term coefficient for “BISS*e-shopping apps” is 0.0725 and passes the 1% significance test. The positive interaction term coefficient suggests that the use of e-shopping apps also plays a positive moderating role.

Life apps (Model 4): The main effect coefficient for lifestyle service app usage is 0.5758, which does not pass the significance test. The interaction term coefficient for “BISS*lifestyle service apps” is 0.0481 and also does not pass the significance test, indicating no significant moderating effect.

Gaming apps (Model 5): The main effect coefficient for gaming app usage is 0.3051, which passes the 1% significance test; however, the interaction term coefficient for “BISS*gaming apps” is 0.0051 and does not pass the significance test, indicating no significant moderating effect.

News apps (Model 6): The main effect coefficient for news and information app usage is −0.2421, which passes the 10% significance test. However, the interaction term coefficient for “BISS*news and information apps” is 0.0664 and does not pass the significance test, indicating no significant moderating effect.

E-book and learning apps (Model 7): The main effect coefficient for e-book and learning app usage is 0.8115, which does not pass the significance test. The interaction term coefficient for “BISS*e-book and learning apps” is 0.0094 and also does not pass the significance test, indicating no significant moderating effect.

Video apps (Model 8): The main effect coefficient for video streaming app usage is 0.9522, which does not pass the significance test. The interaction term coefficient for “BISS*video streaming apps” is 0.0328 and also does not pass the significance test, indicating no significant moderating effect.

In summary, only short video and e-shopping apps play a significant positive moderating role in the relationship between BISS and smartphone addiction among college students. That is, when college students frequently use short video or e-shopping apps, the negative effect of body image satisfaction on smartphone addiction is further intensified. This also means that college students with higher levels of body image dissatisfaction, if they frequently use these two types of apps, will have a significantly higher tendency toward smartphone addiction. This result validates the rationality of Hypothesis 3.

## 6 Discussion

This research examines the addictive behavior of young adults toward smartphones via the lens of dissatisfaction with their body image. This research develops an analytical framework of “self-objectification, body shame, smartphone addiction” grounded in Objectification Theory. This research empirically examines the impact and mechanism of body image dissatisfaction on smartphone addiction among adolescents, utilizing questionnaire survey data from 1,958 college students across 7 universities in 3 provinces of China. The study shows that there is a significant negative correlation between body image satisfaction and smartphone addiction among college students. Specifically, for every 1% decrease in body image satisfaction, the degree of smartphone addiction among college students increases by 5.76%. This finding aligns with the outcomes of other investigations in the current literature. Liu et al. (2023) indicated that an excessive preoccupation with looks among youth can result in frequent engagement with social networking platforms, ultimately leading to internet addiction. Nonetheless, in opposition to the aforementioned finding, Panova and Carbonell (2018) contended that examining contemporary smartphone addiction via the lens of addiction may be unfounded. Certain studies indicate a lack of substantial link with body image dissatisfaction and smartphone usage (Mitchell and Hussain, 2018; Shen et al., 2021). The variability in research findings may stem from the diverse cultural backgrounds of the samples and the inadequate evaluation of the moderating effect of individual psychological resilience. Nonetheless, the most probable neglected element is that the swift evolution and extensive utilization of smartphone technologies have not been considered. The ongoing enhancement and extensive utilization of smartphone content may render prior assessment standards, behavioral manifestations, and influence mechanisms of smartphone addiction inadequate, thereby skewing conclusions.

The heterogeneity analysis revealed gender and discipline-wise variations in the impact of college students’ body image concern on smartphone addiction. From the standpoint of gender disparities, women exhibit a greater susceptibility to smartphone addiction compared to males, aligning with the findings of prior research. Research indicates that female engagement on social media typically surpasses that of males, perhaps due to females’ propensity to utilize social media for social connection and emotional expression (Best et al., 2014; Tifferet, 2019). The impact of body image concern on smartphone addiction is markedly more pronounced among humanities and social sciences students compared to their science and engineering counterparts.

Ultimately, our mechanism analysis revealed that apps serve as a moderating factor in the relationship between body image dissatisfaction and smartphone addiction. The most significant factor is the pervasive use of short-video applications, which intensifies the impact of body image dissatisfaction among college students on smartphone addiction. Short-video applications typically incorporate features aimed at encouraging prolonged user engagement, such as push notifications, reward systems, and personalized suggestions, facilitating the development of habitual usage patterns. Conversely, college students utilizing educational application software exhibit a diminished level of smartphone addiction, perhaps due to the product’s defined objectives and time constraints, which assist users in sustaining concentration (Reynol, 2012). Consequently, subsequent research may investigate how the design attributes of various apps influence user behavior and how to enhance app design to mitigate the risk of smartphone addiction (Kuss and Griffiths, 2011). Simultaneously, educational institutions and parents can assist students in judiciously managing their smartphone usage and advocate for the utilization of apps that are conducive to study and well-being, thus fostering their healthy development (Ganesh et al., 2015). Furthermore, social support and mental health education are crucial strategies for preventing smartphone addiction (Liu and Ma, 2020).

## 7 Research limitations and future prospects

Limitations of Data Format. This study utilizes cross - sectional data for empirical analysis, and this type of data has certain inherent limitations. Since it cannot track changes in individuals over the time dimension, it is difficult to effectively control for individual heterogeneity and time effects, which may limit the accuracy of estimating the relationships between variables. In contrast, panel data regression analysis can better control for individual heterogeneity and time effects, enabling more accurate estimation of relationships. Therefore, future studies may adopt a panel data design, incorporate dynamic changes across the time dimension, and explore the long-term relationships between core variables in greater depth to address the limitations of this study regarding data type.

Limitations of Measurement Tools. This study employed the Smartphone Addiction Scale - Short Version (SAS-SV) and the Body Image State Scale (BISS) to evaluate smartphone addiction and body image satisfaction. Although these scales possess a certain degree of reliability and validity, they may still give rise to measurement errors. For example, both the SAS-SV and BISS are self - reported scales. This implies that participants might respond in accordance with social desirability rather than their actual circumstances. Integrating smartphone usage logs or behavioral tracking technology to gather empirical data would assist in reducing data bias associated with the self - report method.

Limitations of Sample Selection. The sample for this study comprises only 1,958 college students from 7 universities in 3 provinces of China, which may limit the generalizability of the results. If the sample scope is broadened to incorporate participants from more regions with diverse educational and cultural backgrounds, the generalizability of the conclusions will be further improved.

## 8 Conclusion

Smartphone addiction has emerged as a progressively significant issue among college students. During puberty, college students’ preoccupation with body image has progressively heightened, although their excessive quest for an ideal body image has exacerbated their dissatisfaction with their looks. Measuring the extent and mechanism of the impact of young adults’ dissatisfaction with their body image on smartphone addiction is crucial for guiding young adults to effectively address their body image - related discontent and addictive behaviors. This research develops an analytical framework based on objectification theory, encompassing “self-objectification, body shame, and smartphone addiction.” This research empirically examines the impact and mechanism of young adults’ dissatisfaction with their body image on smartphone addiction, utilizing questionnaire survey data from 1,958 college students across 7 universities in 3 provinces in China. The empirical analysis examined the impact and mechanisms of body image dissatisfaction on smartphone addiction among young adults. The results show that there is a significant negative correlation between body image satisfaction and smartphone addiction among college students. Specifically, for every 1% decrease in appearance satisfaction, the degree of smartphone addiction increases by 5.76%.

Moreover, we identified that the impact of body image dissatisfaction on smartphone addiction shows notable variations based on gender and discipline. The marginal effect of body image dissatisfaction on female college students is 1% greater than that on male students, and the marginal effect of body shape anxiety on students in the humanities and social sciences is 2.19% higher than that on students in science and engineering. Subsequent mechanistic analysis reveals that short-video and e-shopping applications have a positive regulatory influence on the correlation between body image dissatisfaction and smartphone addiction. Specifically, the use of short-videos and e-shopping applications significantly amplifies the enhancing effect of individual body image dissatisfaction on smartphone addictive behavior. This provides empirical evidence for alleviating smartphone addiction among adolescents.

## Data Availability

The original contributions presented in the study are included in the article/supplementary material, further inquiries can be directed to the corresponding author.
